# COVID-19: in the absence of vaccination – ‘mask-the-nation’

**DOI:** 10.2217/fmb-2020-0112

**Published:** 2020-07-17

**Authors:** Roy D Sleator, Steven Darby, Alan Giltinan, Niall Smith

**Affiliations:** ^1^Department of Biological Sciences, Cork Institute of Technology, Bishopstown, Cork, Ireland; ^2^Centre for Advanced Photonics & Process Analysis, Cork Institute of Technology, Bishopstown, Cork, Ireland; ^3^Blackrock Castle Observatory, Cork Institute of Technology, Bishopstown, Cork, Ireland

**Keywords:** COVID-19, coronavirus, face masks, SARS, SARS-CoV-2

Coronavirus (CoV) disease 2019 (COVID-19) is a severe respiratory illness first reported in Wuhan, the capital city of Hubei Province, China. The first patient to be hospitalized with COVID-19 was admitted on 12 December 2019 [1]. The symptoms of the disease include fever, an unproductive cough, muscular soreness and dyspnea [2]. Predominantly affecting older people, particularly those with underlying medical conditions, COVID-19 has an estimated mortality rate of 2–5% [3].

The causative agent of COVID-19 is a CoV; named SARS-CoV-2 by the WHO on 11 February 2020 (the same day the disease itself was officially named) [4]. Phylogenetic analysis revealed that the virus is most closely related to a group of SARS-like CoVs (genus *Betacoronavirus*, subgenus *Sarbecovirus*) previously isolated from bats in China [1]. Among this group is SARS-CoV, the causative agent of SARS.

On 11 March 2020, the WHO declared the COVID-19 outbreak a global pandemic [5]. At the time of writing, 213 countries and territories around the world and two international conveyances have reported cases of COVID-19, with the total number surpassing 10 million and over 500,000 associated deaths [6]. While these numbers make for painful reading, the situation could have been significantly worse had it not been for the strict social distancing and isolation measures imposed by most nations in a concerted effort to ‘flatten the curve’ [7]. However, as these measures are eased and at least some sectors of society return to work, we need to consider what procedures are now required to continue protecting a post-lockdown population [8].

In the absence of a vaccine, or effective antiviral, one of our only remaining strategies for controlling COVID-19 is to physically block the spread of SARS-CoV-2 in the community. Given that COVID-19 is a respiratory illness, the most effective physical defense likely involves widespread public use of face coverings, in conjunction with other control measures [9].

Face coverings (also variously referred to as face masks, nonmedical masks, community masks or barrier masks) function primarily in source control; capturing droplets expelled by an infected individual [10]. Droplet spread is widely considered to be the main mode of transmission of SARS-CoV-2 [11]. Small aerosols are created by rupture of bubbles, or thin films, in the bronchioles of the lungs and vocal cords when speaking. Larger droplets >10 μm fail to traverse the 90° bend of the throat, but can be created in the mouth during speech, coughing and sneezing [12]. There is significant uncertainty, to date, as to what size range is most infectious and, even in a single patient, the distribution of SARS-CoV-2 in the respiratory tract appears to vary widely [13]. However, it is known that expelled particles as large as 100 μm can travel more than 2 m in realistic scenarios [14], and due to the much larger volume, can potentially carry a significant viral load and associated infection risk. Once expelled, droplets evaporate to droplet nuclei (which, being ∼tenfold lighter, remain airborne for longer, thus potentially increasing transmission rates [15]).

Despite some early concerns relating to the benefits of public masking in preventing viral spread (specifically influenza virus) [16], a recent meta-analysis by Chu *et al.* [17], involving 172 observational studies across 16 countries and six continents, strongly suggests that face masks reduce the spread of SARS-CoV-2. Macintyre and Chughtai [18] support this view, suggesting that all sectors of society (the community, sick and healthcare workers) will benefit from masking. Indeed, this growing consensus is in line with the findings from the 2003 SARS outbreak in Hong Kong, which show that widespread public use of face masks, together with frequent hand washing and living quarter disinfection, significantly reduced the risk of viral transmission [19]. This, together with evidence from trials with other epidemic respiratory viruses [20], suggests that widespread public masking might be a useful strategy in controlling community spread of SARS-CoV-2.

However, despite this, until recently there has been a reluctance by public health administrators to embrace universal public masking [21]. This reticence centers mainly on two key concerns [9]. First, public demand is likely to lead to even further shortages in already stretched healthcare settings [22]. The second issue relates to potential carelessness and complacency in the general population. While carelessness speaks to inappropriate mask usage (i.e., ill fitting or improperly donned masks), the complacency issue centers on a false sense of security which may accompany mask usage; leading to reduced adherence to other necessary control measures, as previously reported by Yan *et al.* [23].

In line with the most recent public health recommendations [24], and in the absence of an available vaccine or effective antiviral, we suggest that properly designed ‘do it yourself’ (DIY) face masks, fabricated from common household materials, represent the most efficient means of controlling community spread of SARS-CoV-2 (particularly when used in conjunction with appropriate social distancing and hand hygiene practices). DIY face masks reduce demand for medical grade personal protective equipment (PPE) such as N95 masks, thereby safeguarding the medical supply chain and protecting healthcare workers [25]. Furthermore, depending on the materials used, properly designed DIY face masks are often easier to use and more comfortable to wear for prolonged periods. While not as effective as PPE [26], several studies have shown that masks fashioned from common household materials (including tea cloths [27], pillowcases [28] and T-shirts [29]) are at least partially effective in blocking viral spread. Indeed, Zhao *et al.* [30] have helped to quantify the approach using a ranking system (based on filtration quality factor, fabric microstructure and charging ability), to identify the most effective household materials for DIY mask fabrication. Proof of concept for the DIY approach is provided, at least in part, by Ma *et al.*, [31], who have recently shown that homemade masks, composed of four layers of kitchen paper and one layer of cloth, could block 95.15% of the avian influenza virus, compared with 99.98% for N95 masks and 97.14% for surgical masks.

To be effective, everyone, irrespective of whether they are symptomatic or not, should be advised to wear a face covering in public (particularly in situations where appropriate social distancing is either impractical or impossible). This totalitarian approach serves two purposes: first, it overcomes the stigma associated with wearing a mask in public (previously considered by Buregyeya *et al.* [32], in relation to TB patients). Second, it reduces spread by asymptomatic carriers. This is particularly important in the case of SARS-CoV-2, whereby the viral load has been shown to be similar in symptomatic and asymptomatic patients [33], and infection from an asymptomatic contact has already been reported [34]. Furthermore, universal public masking has the added benefit of protecting against other respiratory infectious agents, such as influenza virus [35]. Indeed, co-infection by SARS-CoV-2 and Influenza virus has already been reported in both China [36] and Iran [37]. Encouragingly, and in support of the above, Cowling *et al.* [38] have shown that nonpharmaceutical interventions, including face masks, resulted in a 44% reduction in influenza transmissibility in Hong Kong, in the midst of the current COVID-19 pandemic.

Thus, in the absence of a vaccination strategy, DIY face masks will likely play an important role in stemming the spread of SARS-CoV-2. We conclude by introducing a new idiom to the epidemiology lexicon: in the absence of vaccination, mask-the-nation!
